# Effectiveness of the Standard WHO Recommended Retreatment Regimen (Category II) for Tuberculosis in Kampala, Uganda: A Prospective Cohort Study

**DOI:** 10.1371/journal.pmed.1000427

**Published:** 2011-03-15

**Authors:** Edward C. Jones-López, Irene Ayakaka, Jonathan Levin, Nancy Reilly, Francis Mumbowa, Scott Dryden-Peterson, Grace Nyakoojo, Kevin Fennelly, Beth Temple, Susan Nakubulwa, Moses L. Joloba, Alphonse Okwera, Kathleen D. Eisenach, Ruth McNerney, Alison M. Elliott, Jerrold J. Ellner, Peter G. Smith, Roy D. Mugerwa

**Affiliations:** 1 Section of Infectious Diseases, Department of Medicine, Boston Medical Center, Boston University Medical School, Boston, Massachusetts, United States of America; 2 Makerere University – University of Medicine and Dentistry of New Jersey (UMDNJ) Research Collaboration, Kampala, Uganda; 3 Department of Medicine, New Jersey Medical School –UMDNJ, Newark, New Jersey, United States of America; 4 Medical Research Council–Uganda Virus Research Institute, Uganda Research Unit on AIDS, Entebbe, Uganda; 5 School of Public Health, University of the Witwatersrand, Johannesburg, South Africa; 6 Department of Microbiology, Makerere University College of Health Sciences, Kampala, Uganda; 7 Division of Infectious Diseases, Brigham & Women's Hospital, Harvard Medical School, Boston, Massachusetts, United States of America; 8 Southeastern National Tuberculosis Center, Division of Mycobacteriology, Department of Medicine, University of Florida, Gainesville, Florida, United States of America; 9 Mulago Hospital Tuberculosis Clinic, Mulago Hospital, Kampala, Uganda; 10 Departments of Pathology and, Microbiology and Immunology, University of Arkansas for Medical Sciences, Little Rock, Arkansas, United States of America; 11 Departments of Infectious and Tropical Diseases, London School of Hygiene & Tropical Medicine, London, United Kingdom; 12 Department of Medicine, Makerere University College of Health Sciences, Kampala, Uganda; 13 Epidemiology and Population Health, London School of Hygiene & Tropical Medicine, London, United Kingdom; Harvard School of Public Health, United States of America

## Abstract

**Background:**

Each year, 10%–20% of patients with tuberculosis (TB) in low- and middle-income countries present with previously treated TB and are empirically started on a World Health Organization (WHO)-recommended standardized retreatment regimen. The effectiveness of this retreatment regimen has not been systematically evaluated.

**Methods and Findings:**

From July 2003 to January 2007, we enrolled smear-positive, pulmonary TB patients into a prospective cohort to study treatment outcomes and mortality during and after treatment with the standardized retreatment regimen. Median time of follow-up was 21 months (interquartile range 12–33 months). A total of 29/148 (20%) HIV-uninfected and 37/140 (26%) HIV-infected patients had an unsuccessful treatment outcome. In a multiple logistic regression analysis to adjust for confounding, factors associated with an unsuccessful treatment outcome were poor adherence (adjusted odds ratio [aOR] associated with missing half or more of scheduled doses 2.39; 95% confidence interval (CI) 1.10–5.22), HIV infection (2.16; 1.01–4.61), age (aOR for 10-year increase 1.59; 1.13–2.25), and duration of TB symptoms (aOR for 1-month increase 1.12; 1.04–1.20). All patients with multidrug-resistant TB had an unsuccessful treatment outcome. HIV-infected individuals were more likely to die than HIV-uninfected individuals (*p*<0.0001). Multidrug-resistant TB at enrolment was the only common risk factor for death during follow-up for both HIV-infected (adjusted hazard ratio [aHR] 17.9; 6.0–53.4) and HIV-uninfected (14.7; 4.1–52.2) individuals. Other risk factors for death during follow-up among HIV-infected patients were CD4<50 cells/ml and no antiretroviral treatment (aHR 7.4, compared to patients with CD4≥200; 3.0–18.8) and Karnofsky score <70 (2.1; 1.1–4.1); and among HIV-uninfected patients were poor adherence (missing half or more of doses) (3.5; 1.1–10.6) and duration of TB symptoms (aHR for a 1-month increase 1.9; 1.0–3.5).

**Conclusions:**

The recommended regimen for retreatment TB in Uganda yields an unacceptable proportion of unsuccessful outcomes. There is a need to evaluate new treatment strategies in these patients.

*Please see later in the article for the Editors' Summary*

## Abstract 2

Prospective evaluation of the effectiveness of the WHO-recommended standardized retreatment regimen for tuberculosis by Edward Jones-López and colleagues reveals an unacceptable proportion of unsuccessful outcomes.

## Editors' Summary

**Background:**

One-third of the world's population is currently infected with *Mycobacterium tuberculosis*, the bacterium that causes tuberculosis (TB), and 5%–10% of HIV-uninfected individuals will go on to develop disease and become infectious. The risk of progression from infection to disease in HIV infected is much higher. If left untreated, each person with active TB may infect 10 to 15 people every year, reinforcing the public health priority of controlling TB through adequate treatment. Patients with a previous history of TB treatment are a major concern for TB programs throughout the world because these patients are at a much higher risk of harboring a form of TB that is resistant to the drugs most frequently used, resulting in poorer treatment outcomes and significantly complicating current management strategies. More then 1 million people in over 90 countries need to be “re-treated” after failing, interrupting, or relapsing from previous TB treatment.

Every year, 10%–20% of people with TB in low- and middle-income countries are started on a standardized five-drug retreatment regimen as recommended by the World Health Organization (WHO). Yet, unlike treatment regimens for newly diagnosed TB patients, the recommended retreatment regimen (also known as the category II regimen) has never been properly evaluated in randomized clinical trials or prospective cohort studies. Rather, this regimen was recommended by experts before the current situation of widespread drug-resistant TB and HIV infection.

**Why Was This Study Done?:**

WHO surveillance data suggest that the retreatment regimen is successful in about 70% of patients, but retrospective studies that have evaluated the regimen's efficacy showed variable treatment responses with success rates ranging from 26% to 92%. However, these studies have generally only assessed outcomes at the completion of the retreatment regimen, and few have examined the risk of TB recurrence, especially in people who are also infected with HIV and so are more likely to experience TB recurrence—an issue of particular concern in sub-Saharan Africa. Therefore, in this study based in Kampala, Uganda, the researchers conducted a prospective cohort study to assess treatment and survival outcomes in patients previously treated for TB and to identify factors associated with poor outcomes. Given the overwhelming contribution of HIV infection to death, the researchers categorized their survival analysis by HIV status.

**What Did the Researchers Do and Find?:**

The researchers recruited consecutive smear-positive TB patients who were admitted to Mulago Hospital, Kampala, Uganda, for the retreatment of TB with the standard retreatment regimen between July 2003 and January 2007. Eligible patients received daily directly observed therapy and after hospital discharge, were seen every month during their 8-month TB-retreatment course. Home health visitors assessed treatment adherence through treatment card review, monthly pill counts, and patient self-report. After the completion of the retreatment regimen, patients were evaluated for TB recurrence every 3 months for a median of 21 months. The researchers then used a statistical model to identify treatment outcomes and mortality HIV-uninfected and HIV-infected patients.

The researchers found that 29/148 (20%) of HIV-uninfected and 37/140 (26%) of HIV-infected patients had an unsuccessful treatment outcome. Factors associated with an unsuccessful treatment outcome were poor adherence, HIV infection, increasing age, and duration of TB symptoms. All patients with multidrug resistant TB, a form of TB that is resistant to the two most important drugs used to treat TB, had an unsuccessful treatment outcome. In addition, HIV-infected subjects were more likely to die than HIV-uninfected subjects (*p*<0.0001), and having multidrug resistant TB at enrollment was the only common risk factor for death during follow-up for both HIV-infected and HIV uninfected patients. Other risk factors for death among HIV-infected patients were CD4<50 cells/ml and no antiretroviral therapy treatment and among HIV-uninfected patients were poor adherence and duration of TB symptoms.

**What Do These Findings Mean?:**

The researchers found that although 70%–80% of patients had a successful treatment outcome on completion of antituberculous therapy (a result that compares well with retrospective studies), the standard retreatment regimen had low treatment response rates and was associated with poor long-term outcomes in certain subgroups of patients, particularly those with multidrug resistant TB and HIV.

These findings indicate that the standard retreatment approach to TB as implemented in low- and middle-income settings is inadequate and stress the importance of a new, more effective, strategies. Improved access to rapid diagnostics for TB drug-resistance, second-line TB treatment, and antiretroviral therapy is urgently needed, along with a strong evidence base to guide clinicians and policy makers on how best to use these tools.

**Additional Information:**

Please access these Web sites via the online version of this summary at http://dx.doi.org/10.1371/journal.pmed.1000427.

- The World Health Organization has information on TB, TB retreatment, and multidrug-resistant TB
- WHO also provides information on TB/HIV coinfection
- The Stop TB Partnership provides information on the global plan to stop TB

## Introduction

Each year, 10%–20% of patients with tuberculosis (TB) in low- and middle-income countries present with previously treated TB and are started on therapy empirically with a standardized five-drug retreatment regimen as recommended by the World Health Organization (WHO). It is estimated that annually more than 1 million people in over 90 countries are treated with the 8-mo regimen (2 mo of streptomycin [S], isoniazid [H], ethambutol [E], rifampicin [R], and pyrazinamide [Z]; 1 mo of R, H, E and Z; and 5 mo of R, H and E [2 SHERZ/1 RHEZ/5 RHE]) after failing, interrupting, or relapsing from prior treatment [1],[2].

Unlike treatment regimens for new TB patients, the currently recommended retreatment regimen (also known as the category II regimen) has never been evaluated for efficacy in randomized clinical trials or prospective cohort studies [3]. The WHO formulated the recommendation to add streptomycin to the four first-line drugs used for initial therapy for empiric TB retreatment from expert opinion in an era prior to the emergence of widespread drug-resistant TB (DR-TB) and prevalent HIV infection [4]. Recently updated WHO guidelines recommend drug-susceptibility testing (DST) prior to retreatment [5] and treatment of confirmed treatment failures and suspected multidrug-resistant (MDR) TB cases with region-specific standardized regimens [6]. However, access to DST and to second-line TB drugs remains poor in high-burden countries and, despite the findings that inadequate regimens amplify drug resistance [3],[7]–[9], the standardized retreatment regimen is still generally the mainstay of national programs in resource-limited settings.

On the basis of WHO surveillance data, retreatment regimens successfully treat approximately 70% of cases [2]. However, findings from retrospective studies [1],[3],[8]–[22] evaluating the efficacy of the retreatment regimen, demonstrate a variable treatment response with success rates ranging from 26% to 92%. Poor performance has been attributed to high background rates of DR-TB [1],[15],[19], prevalent HIV infection [20], and poor treatment adherence [10],[14]. Studies evaluating the efficacy of the retreatment regimen have generally only assessed outcomes at the completion of therapy and few have examined the risk for TB recurrence, especially in HIV-infected people who are likely to be prone to recurrence [23],[24]. With prevalent HIV infection and increasingly recognized DR-TB, there is a risk for these two conditions to converge in sub-Saharan Africa creating a “perfect storm,” as recently suggested. [24].

As part of a larger study to assess a new management strategy for DR-TB in resource-limited settings, we conducted a prospective cohort study in Kampala, Uganda to assess outcomes in previously treated TB patients, with particular reference to the impact of drug resistance on treatment outcomes and survival. We sought to identify factors associated with poor outcomes and separated our survival analysis by HIV status, given the overwhelming contribution of HIV infection to early death, and in order to evaluate the impact of antiretroviral treatment (ART) and CD4 count on mortality among TB/HIV coinfected patients. When we began our study in 2003, Uganda lacked the information, training, and resources required to address the problem of DR-TB. As part of this study, we helped the Uganda National Tuberculosis and Leprosy Programme (NTLP) develop the pillars of a rational and sustainable program to control DR-TB in Uganda, which included the provision of treatment with second-line TB drugs when possible.

## Methods

### Ethics Statement

The AIDS Research Sub-Committee of the Uganda National Council of Science and Technology and the Institutional Review Boards at the University of Medicine and Dentistry of New Jersey and the London School of Hygiene & Tropical Medicine approved the study.

This study was conducted at the 85-bed inpatient TB ward of the NTLP Chemotherapy Centre at Mulago Hospital in Kampala, Uganda. The NTLP Centre serves both as a local treatment clinic (largest in Kampala) and as the national referral centre (approximately one-third of attendees are referral cases). Most (95%) of the retreatment cases seen at the NTLP Centre were hospitalized to facilitate administration of streptomycin when clinically possible; however, patients with suspected or known MDR-TB were managed as outpatients. Uganda is on the WHO list of high-burden countries with a 2007 estimated annual TB (all forms) incidence rate of 330 cases per 100,000; 39% with HIV infection and a prevalence of MDR-TB in new and retreatment TB cases of 0.5% and 4.4%, respectively [2]. The Ugandan NTLP reports 100% coverage by directly observed therapy (DOTS), a case detection rate of 39%, and 70% treatment success rate [2].

All consecutive TB patients hospitalized for retreatment between July 2003 and January 2007 were eligible for inclusion in the study, provided they were 18 years or older and gave written informed consent. Eligible individuals were included in the study if they provided sputum specimens that were positive for acid-fast bacilli (AFB) on smear microscopy and TB was confirmed subsequently by growth of *Mycobacterium tuberculosis* in culture. We used a standardized questionnaire to collect demographic and clinical information. At presentation, participants were classified according to the WHO definitions of prior treatment outcomes (relapse, treatment failure, or default) [5] using information from a previous TB-treatment card (55%) or hospital chart (45%). A postero-anterior chest radiograph was taken on all study participants at baseline and an experienced clinician graded the extent of disease on a four-category ordinal scale. Individuals were offered HIV testing and a CD4+ lymphocyte cell count was measured in HIV-infected patients. All patients were offered TB treatment on the basis of the standard 8-mo WHO-recommended category II retreatment regimen composed of 2 mo of streptomycin (S), rifampicin (R), isoniazid (H), ethambutol (E), and pyrazinamide (Z); 1 mo of R, H, E, and Z; and 5 mo of R, H, and E (2 SRHEZ/1 RHEZ/5 RHE). HIV-infected patients with a CD4+ cell count less than 200 cells/µl were referred for ART according to existing national guidelines [25], though access to ART was limited in Kampala before 2005. Of the 90 individuals eligible for ART according to this criterion, 45 (50%) received treatment; eight patients who did not qualify for ART initially, began treatment with ART later.

Second-line drugs for the treatment of MDR-TB were not available under routine care by the NTLP when the study was initiated. After discussions with collaborators, officials from the Ugandan Ministry of Health, NTLP, WHO, and other stake holders, we agreed to use our study as an opportunity to build the necessary foundations for a sustainable MDR-TB program in Uganda. These included measuring the prevalence of DR-TB, developing the laboratory capacity for standard and rapid DST, obtaining preferentially priced second-line TB drugs, training medical and nursing personnel in MDR-TB management, developing a community-based DOT program, and establishing hospital infection control measures. When second-line drugs became available in 2005, treatment for MDR-TB was begun as a pilot treatment program and phased in as drugs became available and conditions allowed. Divided into two groups, a total of 12 MDR patients received individualized treatment with second-line drugs (seven of them were included in this study and five were not included); the first group of patients began treatment in October 2005 and the second in August 2006. All MDR patients in this study were initially treated with the standard retreatment regimen at the time they first presented to the clinic (before their MDR status was known); they all had an unsuccessful treatment outcome and were included in the survival analysis. The results from individualized treatment with second-line drugs are not included in this analysis.

Patients received daily DOT while admitted to the hospital. After hospital discharge, participants were seen monthly during their 8-mo TB-treatment course, at month 9, and quarterly thereafter for the duration of the study. Patients who did not keep their scheduled visits were traced to their homes. During follow-up visits, home health visitors assessed treatment adherence through treatment card review, monthly pill counts, and patient self-report. Adherence was categorized into a five-level ordinal scale (fully adherent, missed less then 20% of doses, missed about one half of the doses, missed most doses, and not adherent at all). The worst assessment at any visit was taken as the summary measure of adherence for each participant. Because of small numbers of patients in some categories, patients were classified as either “mostly adherent” (consisting of the first two categories) or “missed half or more” (consisting of the remaining three categories). For individuals who died or were lost to follow-up before the month-3 visit, we were unable to assess adherence.

During follow-up, two sputum samples were collected for AFB smear microscopy and culture at each of five predefined time points (months 1, 2, 5, 8, and 12); additional samples were collected at later visits if patients had a productive cough. Following WHO treatment outcome definitions [5], participants were classified as “cured” if they completed treatment and had a negative culture on solid medium at the end of treatment. Individuals were classified as having “completed” treatment if they finished the full 8 mo of therapy and were found to be free of TB symptoms at their first posttreatment follow-up visit, but had no culture results at the end of treatment. They were classified as having an “unknown” outcome if they were lost to follow-up (the majority of these patients were treatment defaulters). Individuals were classified as “treatment failures” if they were culture positive at month 8 or, if they were culture positive at month 5 with no culture at month 8 and we had no confirmation that they were free of TB after the end of treatment. At 8 mo, a “successful” TB-treatment outcome was defined as cure or treatment completion with no evidence of remaining disease, and “unsuccessful” outcome as treatment failure, death during treatment, or unknown outcome. During follow up, individuals were evaluated for TB recurrence and vital status was assessed at the end of scheduled follow-up, which was 24 mo after the initiation of TB treatment, or the close of the study.

### Laboratory Methods

#### TB diagnostics

Sputum specimens were processed with the standard digestion and decontamination method using NALC/Na citrate/NaOH. Centrifuged pellets were resuspended in a phosphate buffer solution and used to prepare smears and cultures on 7H10 agar and liquid media. Sputum smear microscopy was performed using auramine O fluorescent stain and reported according to the US CDC microscopy grading scheme (negative or 1+ to 4+) [26]. Sputum sediments were cultured in either BACTEC 460 or BACTEC MGIT 960 (Becton Dickinson Diagnostic Instrument Systems) according to the manufacturer's recommendations [27],[28]. Confirmation of *M. tuberculosis* complex was determined by either the BACTEC NAP test (Becton Dickinson Diagnostic Instrument Systems) or PCR for IS*6110* as previously described [29]. The number of days from inoculation of 12B media until positive (growth index >30) BACTEC culture (day-to-positive [DTP]) was determined for baseline specimens. Initial *M. tuberculosis* isolates obtained from each patient at the time of recruitment were subjected to DST for S, R, H, E, and Z using BACTEC 460 or MGIT 960. For quality assurance of DST, 245 specimens were also tested at the CDC with over 95% concordance. The radiometric BACTEC 460 was used through July 2006 when it was replaced by the fluorometric MGIT 960. Most (87%) baseline specimens were cultured using the BACTEC 460 system and the majority of the initial isolates tested for drug susceptibility with the 460. The isolation rate and DTP liquid culture for baseline specimens are comparable with the BACTEC 460 and MGIT 960 systems. Likewise the DST results are highly correlative between the two systems. Thus, the change in methodologies during the study had no impact on the interpretation of results.

#### Strain genotyping

We stored the baseline isolate for each participant and all isolates from patients who had a culture positive for *M. tuberculosis* at any time after completing treatment. For each patient with TB recurrence, we compared the patient's initial and recurrent isolates using IS*6110* restriction fragment length polymorphism (RFLP) analysis to differentiate participants with relapse (identical isolates) from those with reinfection (different isolates).

#### HIV testing

HIV testing was offered to all participants; HIV infection status was determined using a serial rapid test algorithm in concordance with international guidelines [30]. Determine (Abbott Laboratories, Abbott Japan Co. Ltd) was used for screening with positive results confirmed by Statpack (Chembio Diagnostic Systems). Samples with discordant results were examined using Unigold (Trinity Biotech PLC). Rare samples positive by Determine, but negative by Statpack and Unigold, were resolved by PCR.

### Statistical Analysis

The objectives of the statistical analysis were to describe the treatment outcomes and mortality of HIV-uninfected and HIV-infected patients and to find factors associated with treatment outcome and mortality. Logistic regression was used to identify factors associated with unsuccessful treatment outcome. Factors were considered for inclusion as risk variables or confounders in models on substantive grounds, i.e., on the basis of prior knowledge or biological plausibility [31]. For continuous explanatory variables, nonlinearity was tested through the fitting of fractional polynomials [32]. In order to produce a more parsimonious model, variables were excluded from the final model, in a backward elimination type of approach, if they were not significant at the 15% significance level (in the case of confounders) or at the 5% significance level in the case of risk factors, and were not deemed to be sufficiently important to be retained in the model. In addition, the inclusion of interaction terms between HIV and the other risk factors was investigated.

Survival analysis methods were used to investigate factors associated with mortality. Kaplan Meier survival curves were derived to compare mortality between HIV-infected and HIV-uninfected patients, and to evaluate the impact of baseline CD4 count and ART treatment on mortality among those HIV-infected. Cox proportional hazards models were fitted to find factors associated with mortality. Separate models were fitted for HIV-uninfected and HIV-infected individuals to allow for inclusion of CD4+ cell count and ART-status in the model for HIV-infected individuals. A composite five-level variable for CD4+ cell count and ART was constructed (CD4≥200, CD4 50–199+no ART, CD4 50–199+ART, CD4<50+no ART, and CD4<50+ART), because of the strong correlation between CD4+ cell count and ART. The composite variable of CD4+ cell count and ART use was analyzed as a time-varying covariate as individuals initiated ART at different times during follow-up. Baseline CD4+ cell count was used for all analyses. The proportional hazards assumption was checked using graphical methods as well as the test of Grambsch and Therneu [33]. All analyses were conducted using Stata, release 10.1.

## Results

### Study Patients

A total of 288 eligible patients, 148 (51%) HIV-uninfected and 140 (49%) HIV-infected, were enrolled in the study between July 2003 and January 2007. Their baseline characteristics are summarized by HIV status in Table 1. HIV-uninfected patients were more likely to be male, and to have advanced disease and cavitations on chest radiograph. HIV-infected patients were older, presented with a higher average Karnofsky score, and presented more frequently with constitutional (fevers, rigors, loss of appetite, malaise, and weight loss) symptoms (not shown in Table 1). The most common reason for retreatment in HIV-uninfected patients was defaulting from prior treatment, whereas for patients with HIV it was for TB relapse. At the time of enrolment, the median CD4+ cell count in HIV-infected patients was 120 cells/µl (interquartile range [IQR] 34–287 cells/µl) and 12 (9%) were receiving treatment with ART.

**Table 1:** Baseline characteristics of 288 hospitalized retreatment TB patients in Kampala, Uganda, according to HIV infection status.

Characteristic	Level	*n* HIV Uninfected (%)^a^ (*n* = 148)	*n* HIV Infected (%)^a^ (*n* = 140)	*p*-Value^b^
Age (y)	<30	84 (57)	43 (31)	
	30–40	37 (25)	67 (48)	<0.001
	>40	27 (18)	30 (21)	
	Mean (SD)	32.6 (11.0)	35.4 (8.6)	0.016
Sex	Female	29 (20)	64 (46)	<0.001
	Male	119 (80)	76 (54)	
Karnofsky Score	≥70	138 (93)	118 (84)	0.016
	<70	10 (7)	22 (16)	
Body mass index (kg/m^2^)	<15	21 (14)	14 (10)	
	15–18.5	66 (45)	80 (58)	0.11
	>18.5	59 (40)	45 (32)	
	Mean (SD)	17.9 (2.7)	17.9 (2.5)	0.93
Reason for TB retreatment	Relapse	67 (45)	83 (59)	
	Failure	6 (4)	3 (2)	0.052
	Defaulter	75 (51)	54 (39)	
Duration of cough prior to enrollment (wk)	Median (IQR)	12 (8–20)	12 (6–16)	0.16
Duration of TB symptoms	<3 mo	93 (63)	102 (73)	
	3–6 mo	35 (24)	26 (19)	0.17
	>6 mo	20 (14)	12 (9)	
	Median (wk) (IQR)	12 (8–20)	11 (6–16)	0.12
Number of previous TB episodes	1	128 (86)	114 (81)	
	2	15 (11)	19 (14)	0.49
	≥3	5 (3)	7 (5)	
Time since most recent TB episode (mo)	Median (IQR)	14 (9–29)	16 (10–37)	0.46
Drug resistance status	Sensitive to H and R	118 (80)	116 (83)	
	Resistance to H or R	19 (13)	17 (12)	0.67
	MDR	11 (7)	7 (5)	
Extent of disease on chest radiograph	Normal	0	9 (7)	
	Minimal	7 (5)	16 (12)	<0.001
	Moderate	29 (20)	48 (35)	
	Severe	111 (76)	63 (46)	
Cavitary disease		121 (82)	70 (51)	<0.001
Miliary infiltrate		4 (3)	3 (2)	0.78
Sputum AFB smear microscopy grade	1+	2 (1)	5 (4)	
	2+	8 (5)	15 (11)	0.16
	3+	19 (13)	21 (15)	
	4+	119 (80)	99 (70)	
Days-to-positive culture (BACTEC)	≥7 d	40 (27)	40 (29)	0.77
	<7 d	108 (73)	100 (71)	
CD4 cell count (cells/µl)^c^	≥350	—	27 (19)	
	200–349	—	22 (16)	
	50–199	—	44 (32)	n/a
	<50	—	46 (33)	
	Median (IQR)	—	120 (34–287)	

a Unless specified.

b *p*-Values based on a chi-squared test for categorical variables and a *t*-test for continuous variables.

c One HIV-infected participant did not have CD4 result.

AFB, acid-fast bacilli; CD4, CD4+ T lymphocytes; H, isoniazid; R, rifampicin; n/a, not available; SD, standard deviation.

### Baseline Drug Resistance

66 (23%) participants had an *M. tuberculosis* isolate that was resistant to at least one first-line antituberculosis drug: 18 (6%) had MDR, 34 (12%) had mono-resistance to isoniazid, and two (1%) individuals had mono-resistance to rifampicin. The remaining 12 (4%) individuals had mono-resistance to streptomycin (eight), ethambutol (three), or pyrazinamide (one). The proportion of individuals with MDR-TB at baseline was similar for HIV-uninfected and HIV-infected patients, 11 (7%) and seven (5%), respectively (*p* = 0.40).

### Follow-up of Study Participants

**Figure 1:**
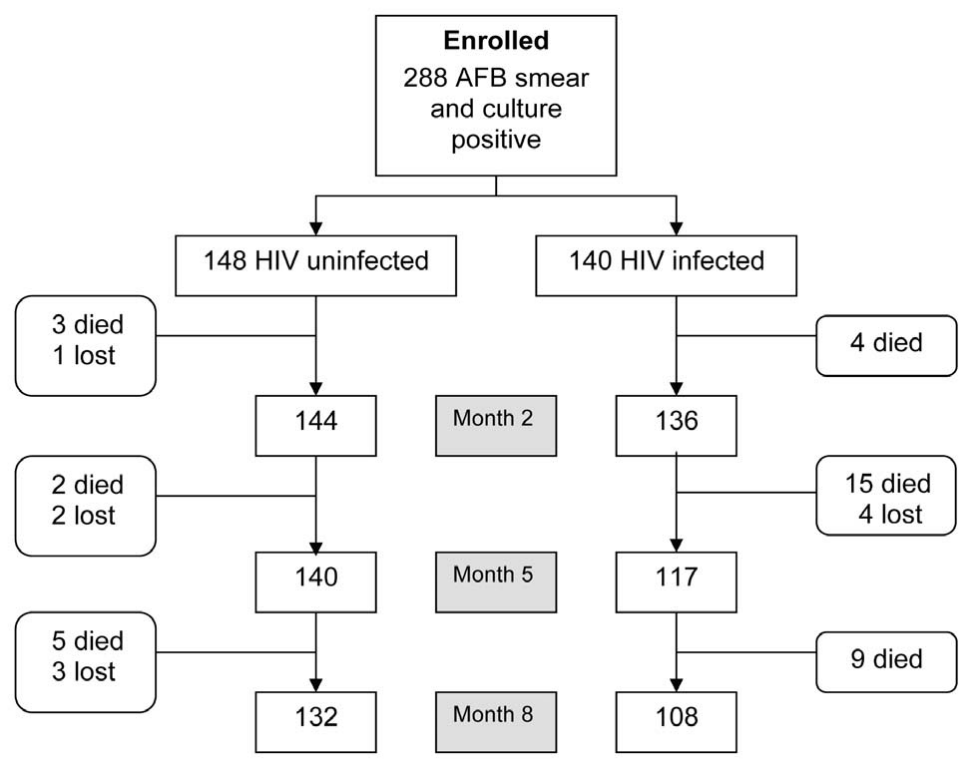
Study profile during the 8-mo TB retreatment period.

Follow-up results during the 8-mo TB-treatment phase are summarized by HIV status in Figure 1. After completing TB treatment, the 240 (83%) individuals retained in the treatment cohort were followed for a period between 1 and 42 mo, median 21 mo (IQR 12–33 mo). In total, 68 (24%) individuals were lost to follow-up, 42 (28%) of these were HIV-uninfected individuals and 26 (19%) of these were HIV-infected individuals. The remaining patients either (a) completed a total of 24 mo of follow-up (i.e.,16 mo post-TB treatment), (b) were seen at a follow-up visit during the last 3 mo of the study, or (c) were recorded as having died during the study.

### Treatment Adherence

Patients were admitted for the 2-mo course of injectable streptomycin treatment for a median duration of 57 d (IQR 53–61 d), during which time all patients had daily DOT. After discharge, 217 (75%) individuals were fully adherent or missed a few doses (less than 5% of individuals) and 59 (21%) missed half or more of their doses. Adherence could not be measured in 11 (4%) patients as they either died or were lost to follow-up before the month-3 visit. Adherence to TB treatment after hospital discharge did not differ between HIV-uninfected (77%) and HIV-infected patients (74%) (*p* = 0.64).

### TB-Treatment Outcomes and Recurrence

We assessed treatment outcomes at the scheduled completion of TB therapy and also followed participants for TB recurrence. At 8 mo (Table S1), 29 HIV-uninfected (20%) and 37 HIV-infected (26%) patients had an unsuccessful treatment outcome (i.e., died during treatment, failed TB treatment, or unknown outcome). All 18 individuals with MDR-TB had an unsuccessful treatment outcome: seven died, nine failed treatment, and two had an unknown outcome at 8 mo. In a logistic regression model (Table 2), an unsuccessful treatment outcome was associated with older age, HIV infection, poor adherence, a longer duration of TB symptoms, and a history of treatment failure. There was no evidence that mono-resistance to isoniazid (INH) was associated with an unsuccessful treatment outcome or of an interaction between HIV and any other risk factor.

**Table 2:** Results of fitting multiple logistic regression models for factors associated with unsuccessful treatment outcome (*n* = 288).

Factor	Level	Adjusted Odds Ratio^a^	95% Confidence Limits	*p*-Value
Age	10-y increase	1.69	1.19–2.41	0.004
HIV status	Uninfected	1		
	Infected	2.44	1.11–5.36	0.026
Baseline resistance status	Sensitive to H and R	1		
	Resistant to H or R	1.63	0.61–4.36	0.33
	MDR	See footnote b		
Reason for TB retreatment	Relapse	1		
	Failure	26.18	1.32–517.7	0.032
	Defaulter	1.27	0.59–2.73	0.54
Treatment adherence	Mostly adherent^c^	1		
	Missed half or more	2.43	1.09–5.40	0.030
Duration of TB symptoms	1-mo increase	1.12	1.04–1.20	0.003

a Odds ratios adjusted for other terms in the model including HIV status, drug resistance status, duration of TB symptoms, age, sex, adherence, and duration since last TB episode.

b All patients with MDR had an unsuccessful outcome so the odds ratio cannot be estimated; 18 observations were thus omitted from the model.

c Category includes participants that were fully adherent with treatment and those that missed a few doses.

H, isoniazid; R, rifampicin.

A total of 222 (77%) individuals were studied for TB recurrence (i.e., patients who did not fail treatment or die during the treatment period and were not lost to follow-up during or immediately after the 8-mo treatment period); of these, 13 (6%) had recurrent TB (seven HIV-uninfected, six HIV-infected), a TB recurrence rate of 4.2 per 100 person-years of observation (PYO). Among the 12 patients with recurrent TB and available RFLP results, seven (58%) had reinfection (two HIV-uninfected and five HIV-infected) and five (42%) had relapse (five HIV-uninfected, one of whom had isoniazid mono-resistance at baseline and one HIV-infected).

### Survival

**Figure 2:**
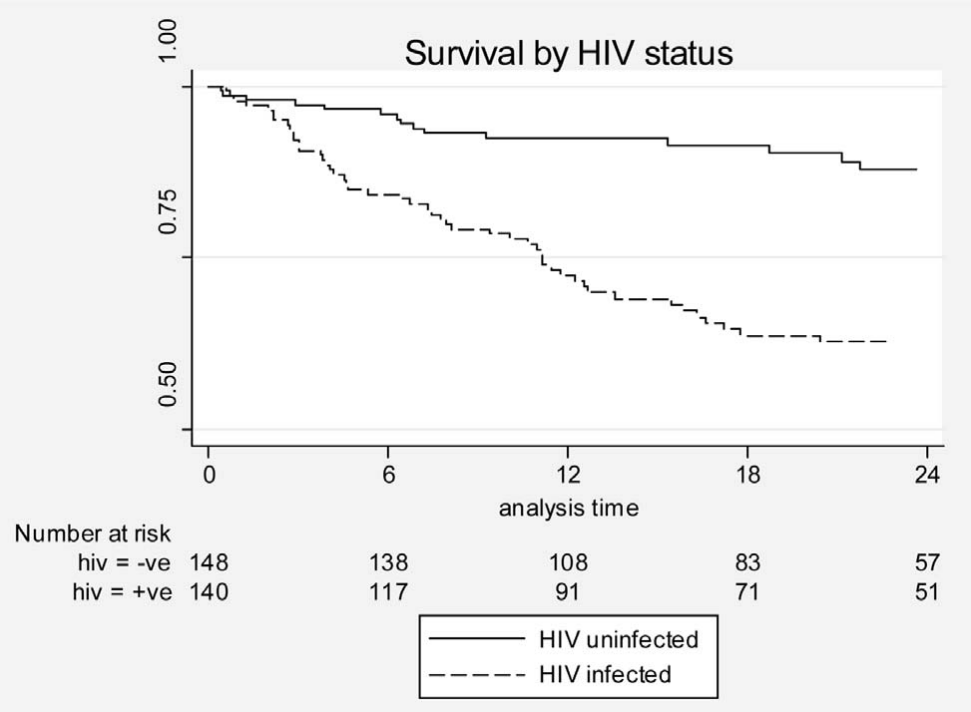
Kaplan-Meier curves by HIV status.

Mortality was analyzed from initiation of antituberculous treatment until the end of follow-up for all patients. In total, 67 patients (23%) died during 511 PYO; 38 (13%) individuals died during the 8-mo TB-treatment course (Figure 1) and 29 (10%) after completing treatment (six HIV-uninfected, 23 HIV-infected). HIV-infected participants (mortality rate 21.4 per 100 PYO) were more likely to die (*p*<0.0001) than HIV-uninfected (5.9 per 100 PYO) (Figure 2). Of the 90 HIV coinfected patients who started the study with CD4 cell counts below 200 cells/µl, 45 (50%) were able to access ART before the end of the study; 30 (67%) patients who did not access ART died compared to eight (18%) who did access ART.

In unadjusted analyses (Table S2), a history of treatment failure, longer duration of symptoms, and MDR at enrolment were all factors associated with an increased risk of death in patients with and without HIV infection; other risk factors for mortality differed between the two groups. In multivariable analyses (Table 3), the presence of MDR at the time of enrollment was (a) the only characteristic common to both HIV-infected and HIV-uninfected patients and (b) the strongest predictor of mortality (an approximately 15-fold increase in risk of death). Other factors associated with death in HIV-uninfected individuals were poor treatment adherence and longer duration of symptoms prior to enrollment. For HIV-infected individuals older age, a lower Karnofsky score, and having a baseline CD4<50 cells/µl and not initiating ART were also associated with an increased risk of death.

**Table 3:** Mortality: results of fitting Cox proportional hazards regression models by HIV status.

Factor	Level	HIV Uninfected (*n* = 148)^a^		HIV Infected (*n* = 140)^b^	
		Hazard Ratio (95% CI)	*p*-Value	Hazard Ratio (95% CI)	*p*-Value
Resistance status	Sensitive to H and R	1	—	1	
	Resistant to H or R	2.2 (0.4–11.1)	0.33	1.4 (0.6–3.3)	0.41
	MDR	14.7 (4.1–52.2)	<0.001	17.9 (6.0–53.4)	<0.001
Duration of TB symptoms	1-mo increase	1.9 (1.0–3.5)	0.05	—	—
TB-treatment adherence	Mostly adherent^c^	1		—	—
	Missed half or more	3.5 (1.1–10.6)	0.027	—	—
Age	10-y increase	—	—	1.4 (0.99–1.9)	0.059
Karnofsky score	≥70	—	—	1	
	<70	—	—	2.1 (1.1–4.1)	0.019
Baseline CD4 (cells/µl) and ART use	CD4≥200	n/a	n/a	1	
	CD4 50–199, no ART	—	—	1.9 (0.7–5.0)	0.17
	CD4 50–199, ART	—	—	1.2 (0.3–4.6)	0.80
	CD4<50, no ART	—	—	7.4 (3.0–18.8)	<0.001
	CD4<50, ART	—	—	2.1 (0.7–6.7)	0.20

a For HIV-uninfected participants, the potential risk factors considered were resistance status, adherence to TB treatment, duration of TB symptoms, reason for being retreated, and BACTEC days to positivity at baseline, with age and sex as potential confounders.

b For HIV-infected participants, the potential risk factors and confounders considered were as for HIV-uninfected with in addition a composite factor measuring baseline CD4 count and ART status; this factor has five levels namely “CD4≥200,” “CD4 50–199+no ART,” “CD4 50–199+ART,” “CD4<50+no ART,” and “CD4<50+ART.”

c Category includes participants who were fully adherent with treatment and those that missed a few doses.

CI, confidence interval; H, isoniazid; n/a, not available; R, rifampicin.

## Discussion

In this prospective cohort study of previously treated pulmonary TB patients managed under programmatic conditions in Uganda, we found that 70%–80% of patients with and without HIV coinfection had a successful treatment outcome at the conclusion of antituberculous therapy (i.e., cured and completed treatment). Although this overall response compares well with many other studies, we found that the retreatment regimen has unacceptably low treatment response rates in certain subgroups of patients and is associated with poor long-term outcomes, particularly in MDR-TB and in HIV-infected patients. Initiation of treatment with ART in eligible patients mitigated much of the excess mortality associated with HIV infection alone, in agreement with a recent clinical trial showing that use of ART concurrently with antituberculous therapy in TB patients is safe and improves survival [34]. HIV-infected patients with MDR-TB had the highest death rate, but we could not ascertain if death was a consequence of advanced HIV disease, TB, or both.

In accordance with the recent review by Menzies and colleagues [3], this is the first prospective study to evaluate the effectiveness of the standardized retreatment regimen using accepted outcome definitions [35]. Despite its use for more than three decades in over 90 countries, there is growing concern that this therapeutic strategy leads to amplification of drug resistance and poor treatment outcomes [1],[3],[23],[36]. Previous studies were limited by their retrospective design and use of unstandardized outcome measures [35]. Our use of programmatic end-points backed by culture results overcomes the potential biases inherent in retrospective studies. The 70%–80% treatment success rate we observed is broadly in agreement with results from the 1980s [4], a large multiyear (1996–2003) study from Morocco [15], and a small recent study from Turkey [21]. However, these results are significantly better than much of the published evidence [1],[10],[12]–[14],[16],[19],[20],[22].

This wide variability is mainly explained by differences in the relative frequency of three prognostic factors across study populations, which include the levels of treatment adherence, MDR-TB, and HIV infection. Among these factors, MDR-TB seems to have a prominent influence on the efficacy of the retreatment regimen as shown in the ecological study by Mak and colleagues [1], where the treatment success rate was significantly decreased as the prevalence of MDR reached a tipping point of 3%, beyond which the failure rate doubled from 5% to 10.6% (*p*<0.0001). In this study, all patients with MDR, especially those with HIV, not surprisingly did poorly given the composition of the retreatment TB regimen. However, comparatively few patients had baseline MDR and thus, the overall attributable risk of MDR to poor TB-treatment outcomes or death was only 27%. Furthermore, other forms of DR-TB such as mono-resistance to isoniazid or rifampicin, present in approximately 20% of the cohort, did not predict treatment outcome or survival.

Taken together, the available data show that the retreatment regimen is effective in treatment of adherent patients with pan-susceptible or mono-resistant *M. tuberculosis*, resulting in acceptable clinical outcomes in most retreatment cases at the conclusion of TB therapy. However, despite these relatively short-term optimistic findings, this cohort of retreatment TB patients experienced poor long-term outcomes. In this sub-Saharan African programmatic setting, of the patients studied for TB recurrence after satisfactory completion of TB treatment, 6% had TB recurrence while a further 9% died, and 18% were lost to follow-up. In addition seven patients who had an unsatisfactory outcome at month 8 (either treatment failure or unknown outcome) later died. This finding, which has been previously reported [17],[23], but is infrequently discussed in the literature, suggests caution when interpreting standard success rates determined at the conclusion of TB treatment. Furthermore, although limited by small numbers, our RFLP results suggest caution in the continued programmatic use of the term “relapse” to describe all patients with TB recurrence and, that strategies to prevent recurrence after completing treatment may be different for HIV-uninfected (mostly relapses) and HIV-infected (mostly reinfection) TB patients in Uganda. Further research is needed to understand the determinants of long-term prognosis in TB patients in sub-Saharan Africa and evaluate which, if any, of the known risk factors associated to poor outcomes are modifiable.

The poor prognosis of retreatment TB patients is usually ascribed to undiagnosed DR-TB and this is leading to a long overdue improvement in laboratory and treatment capacity in resource-limited settings. However, there is a need to further understand if the rapid death (within 1–3 wk after presentation) observed in some patients with HIV/AIDS and DR-TB is a consequence of advanced HIV disease, suboptimally treated TB, or both [37],[38]. In addition, further outcomes-based studies are required to evaluate the efficacy, safety, and cost-effectiveness of early detection of DR-TB using rapid DST testing methods in populations with variable rates of DR-TB and HIV/AIDS. It is likely that the best strategy will involve rapid and adequate treatment of both DR-TB (with new, more effective treatment regimens) and HIV disease [34]. Lastly, our results add to the large body of evidence showing that the provision of DOT should be a major priority in any TB-treatment strategy.

At the annual collaborator's meetings in Kampala, our group had long and impassioned discussions regarding one very difficult ethical dilemma we faced: in a setting such as Uganda where second-line TB drugs are not routinely available, can research in MDR-TB be ethically sound? Although a profound debate on this issue would be beyond the scope of this manuscript, we believe it is important to provide the reader with a summary of our thinking and our actions. Our discussions focused on possible alternatives to our study design and the provision of treatment with second-line drugs considering the time, cost, and complexities involved in building an MDR-TB–treatment program. We considered three alternate designs. Excluding known or suspected MDR patients (and other resistance patterns that are likely to fail treatment or relapse with the retreatment regimen) would have significantly reduced the generalizability of our results and excluded the very population that “exposes” the limitations of the regimen we intended to evaluate. We also considered withholding DST results performed at enrolment, but we reasoned this would have only artificially lessened the ethical concerns we had. Finally, we considered discontinuing the study of MDR-TB patients at the time their DST results became available. During our discussions, it became evident that none of these options would have improved the care of study participants and rather, would prevent them from the benefits of research participation, including the eventual access of some of them to second-line drugs. Even before second-line drugs became available through our program, we are confident patients benefited from study participation both directly (improved treatment and reduced toxicity) and indirectly (patient education and reduced transmission) when compared to existing standard of care in Uganda. With no access to second-line drugs and a prevalence three times higher then expected [36], we were surprised and concerned at the amount of MDR-TB that we discovered, and we immediately tried to find ways to treat these patients. Benefiting from the significant expertise and knowledge on MDR-TB of collaborators and consultants attending our meetings, our group carefully considered the best strategy to initiate and coordinate an MDR-TB–treatment program with the NTLP. At that time, we were unaware of any other MDR-TB programs in sub-Saharan Africa (other than those in South Africa) to help guide our actions. One option we considered was to focus on the immediate, yet costly and likely unsustainable provision of MDR-TB–treatment using imported personnel, technologies, and drugs. This approach, in fact, led to effective community mobilization to sustain an MDR-TB program in Peru [39]; however, Uganda differs from Peru as it has a lower burden of TB and of HIV and almost 10-fold higher gross domestic product (GDP) per capita. An alternate strategy we debated was to place priority on building, from the ‘bottom up’, the necessary foundations for a sustainable program, while treating as many MDR-TB patients as possible. Although we recognize neither of these options is ideal, we decided to pursue the second option because of the input from Ugandan collaborators attending our annual meetings; as the study progressed and data became available, our decisions were based on consensus and compromise. The challenges we faced were enormous, but these are not limited to Uganda as even in 2010, it is estimated that less than 2% of MDR-TB patients in the world have access to adequate treatment [40],[41]. Our hope is that our data-driven call to improve MDR-TB management acts as a powerful complement to two recent editorials advocating for such a change [40],[41].

Our results should be interpreted in the context of the study design and its limitations. Our design may have biased findings towards improved outcomes when compared to true programmatic conditions for several reasons. Firstly, because the clinic attempted to manage patients with suspected MDR-TB as outpatients in an effort to reduce nosocomial transmission, this study did not include all MDR-TB patients diagnosed at this site during the study period. Inclusion of the entire cohort of MDR-TB patients we have reported elsewhere [36], would have decreased the treatment response rate of this study. Secondly, given that this study only included hospitalized patients with daily DOT during the intensive treatment phase and improved follow-up after discharge (presumably resulting in improved treatment outcomes), the inclusion of all retreatment TB patients at this site would have surely resulted in more unfavorable outcomes. Finally, our study required patients to be smear and culture positive; the inclusion of smear-negative individuals, which is more frequently seen in advanced HIV-infected patients, may have lowered the treatment success rate of this cohort. Also, all deaths in the study were considered as unfavorable outcomes of TB retreatment. We did not determine the cause of death of individuals who died and this may have inflated the mortality associated with retreatment, particularly for HIV-infected patients. Also, after completion of TB treatment, retention dropped with approximately 20% of patients lost to follow-up after completing TB treatment. The analysis assumed that these patients had the same survival experience as other patients; however, on the basis of our prior experience, it is probable that the population lost to follow-up experienced greater mortality. If true, the actual mortality may be greater than estimated due to this differential loss. We believe, however, our results provide a true reflection of outcomes in a programmatic setting in sub-Saharan Africa and none of these factors would invalidate the conclusions of this study.

The results of this prospective study provide evidence that the standard retreatment approach to TB, as implemented in low- and middle-income settings, is inadequate. Improved access to rapid diagnostics for TB drug resistance, second-line TB treatment, and antiretroviral therapy is urgently needed, but the evidence base guiding the utilization of these tools by clinicians and planners remains sparse. As has been advocated [1],[7],[42], clinical trials of new approaches to retreatment TB in areas heavily affected by HIV and TB should be a priority. Our findings indicate the importance of a new, more effective strategy for the management of DR-TB in low- and medium-income settings with prevalent HIV infection.

## Supporting Information

Table S1 Clinical outcomes according to HIV and potential prognostic variables after completing the standard 8-mo retreatment regimen in 288 patients in Kampala, Uganda, 2003–2007.
(0.09 MB PDF)

Table S2 Mortality by HIV status and potential explanatory factors.
(0.09 MB PDF)

## Abbreviations

ART: antiretroviral treatment
DR: drug resistant
DST: drug-susceptibility testing
IQR: interquartile range
MDR: multidrug resistant
NTLP: [Uganda] National Tuberculosis and Leprosy Programme
PYO: person-years of observation
RFLP: restriction fragment length polymorphism
TB: tuberculosis
WHO: World Health Organization
