# Supplementary material for: Effectiveness of the Standard WHO Recommended Retreatment Regimen (Category II) for Tuberculosis in Kampala, Uganda: A Prospective Cohort Study
Source: PLoS Med. 2011 Mar 15;8(3):e1000427. doi: 10.1371/journal.pmed.1000427 (PMC3058098; doi:10.1371/journal.pmed.1000427)
Supplement: Table S1 — Clinical outcomes according to HIV and potential prognostic variables after completing the standard 8-mo retreatment regimen in 288 patients in Kampala, Uganda, 2003–2007. (0.09 MB PDF) [file pmed.1000427.s001.pdf]

Table S1: Clinical outcomes according to HIV and potential prognostic variables after completing the standard 8-month retreatment regimen in 288 patients in Kampala, Uganda – 2003 to 2007.

Study Group		Treatment Outcome Category – n (%) –				
		Cured / Completed	Died before Month 8	Failed	Unknown	Totals
**HIV uninfected**		119 (80)	10 (7)	13 (9)	6 (4)	148
Age (years)	< 30	70 (83)	4 (5)	8 (10)	2 (2)	84
	30-40	29 (78)	3 (8)	3 (8)	2 (5)	37
	> 40	20 (74)	3 (11)	2 (7)	2 (7)	27
Sex	Female	22 (76)	3 (10)	4 (14)	0	29
	Male	97 (81)	7 (6)	9 (8)	6(5)	119
Karnofsky score	≥ 70	110(80)	9 (7)	13 (9)	6 (4)	138
	< 70	9 (90)	1(10)	0	0	10
Resistance status	Sensitive to H and R	103 (87)	7 (6)	4 (3)	5 (4)	118
	Resistant to H or R	16 (74)	1 (5)	1 (5)	3 (16)	19
	MDR	0	2 (18)	8 (73)	1 (9)	11
Adherence in continuation phase	Mostly adherent[1]	96 (84)	4 (3)	12 (11)	2 (2)	114
	Missed half or more	23 (77)	3 (10)	1 (3)	3 (10)	30
	No information	0	3 (75)	0	1 (25)	4
Duration of TB symptoms (months)	< 3 months	81 (87)	3 (3)	6 (6)	3 (3)	93
	3-6 months	25 (71)	4 (11)	3 (9)	3 (9)	35
	> 6 months	13 (65)	3 (15)	4 (20)	0	20
Body mass index kg/m^2 (grouped)	< 15	16 (76)	2 (10)	2 (10)	1 (5)	21
	15-18.5	58 (88)	2 (3)	5 (8)	1 (2)	66
	> 18.5	44 (75)	5 (9)	6 (10)	4 (7)	59
Reason for Retreatment	Relapse	57 (85)	4 (6)	5 (7)	1 (1)	67
	Treatment failure	1 (17)	1 (17)	4 (67)	0	6

	Defaulter	61 (81)	5 (7)	4 (5)	5 (7)	75
Extent of disease on chest radiograph	Minimal	6 (86)	1 (14)	0	0	7
	Moderate	22 (76)	0	7 (24)	0	29
	Severe	90 (81)	9 (8)	6 (5)	6 (5)	111
Cavitation present	No	20 (77)	2 (8)	3 (12)	1 (4)	26
	Yes	98 (81)	8 (7%	10 (8)	5 (4)	121
Sputum AFB smear grade	1+ / 2+	6 (60)	4 (40)	0	0	10
	3+	17 (90)	0	1 (5)	1 (5)	19
	4+	96 (81)	6 (5)	12 (10)	5 (4)	119
BACTEC days to positivity (days)	≥ 7	34 (85)	3 (7.5)	3 (7.5)	0	40
	< 7	85 (79)	7 (7)	10 (9)	6 (6)	108
**HIV infected**		103 (74)	28 (20)	5 (4)	4 (3)	140
Age (years)	< 30	31 (72)	6 (14)	3 (7)	3 (7)	43
	30-40	54 (81)	12 (18)	0	1 (1)	67
	> 40	18 (60)	10 (33)	2 (7)	0	30
Sex	Female	45 (70)	14 (22)	3 (5)	2 (3)	64
	Male	58 (76)	14 (18)	2 (3)	2 (3)	76
Karnofsky score	> 70	90 (76)	19 (16)	5 (4)	4 (3)	118
	≤ 60	13 (59)	9 (41)	0	0	22
Resistance status	Sensitive to H and R	94 (81)	18 (16)	2 (2)	2 (2)	116
	Resistant to H or R	9 (53)	5 (29)	2 (12)	1 (6)	17
	MDR	0	5 (71)	1 (14)	1 (14)	7
Adherence in continuation phase	Mostly adherent[1]	85 (82)	13 (13)	2 (2)	3 (3)	103
	Missed half or more	18 (62)	7 (24)	3 (10)	1 (3)	29
	No information	0	8 (100)	0	0	8
Duration of TB symptoms (months)	< 3 months	79 (77)	20 (20)	3 (3)	0	102
	3-6 months	18 (69)	5 (19)	2 (8)	1 (4)	26

	> 6 months	6 (50)	3 (25)	0	3 (25)	12
Body mass index kg/m^2 (grouped)	< 15	10 (71)	4 (29)	0	0	14
	15-18.5	58 (72.5)	18 (22.5)	2 (2.5)	2 (2.5)	80
	> 18.5	34 (76)	6 (13)	3 (7)	2 (4)	45
Reason for Retreatment	Relapse	62 (75)	18 (22)	2 (7)	1 (1)	83
	Treatment failure	0	2 (67)	0	1 (33)	3
	Defaulter	41 (76)	8 (15)	3 (6)	2 (4)	54
Extent of disease on chest radiograph	Normal	6 (67)	3 (33)	0	0	9
	Minimal	14 (88)	2 (12)	0	0	16
	Moderate	35 (73)	9 (19)	2 (4)	2 (4)	48
	Severe	46 (73)	13 (21)	3 (5)	1 (2)	63
Cavitation present	No	48 (73)	15 (23)	1 (2)	2 (3)	66
	Yes	53 (76)	12 (17)	4 (6)	1 (1)	70
Sputum AFB smear grade	1+ / 2+	15 (75)	5 (25)	0	0	20
	3+	17 (81)	3 (14)	1 (5)	0	21
	4+	71 (72)	20 (20)	4 (4)	4 (4)	99
BACTEC days to positivity (days)	≥ 7	32 (78)	7 (17.5)	0	1 (2.5)	40
	< 7	71 (72)	21 (21)	5 (5)	3 (3)	100

H= Isoniazid R= Rifampicin MDR= Multi-drug resistant AFB= Acid-fast bacilli

[1] Category includes subjects that were fully adherent with treatment and those that missed a few doses
