# Supplementary material for: Effectiveness of the Standard WHO Recommended Retreatment Regimen (Category II) for Tuberculosis in Kampala, Uganda: A Prospective Cohort Study
Source: PLoS Med. 2011 Mar 15;8(3):e1000427. doi: 10.1371/journal.pmed.1000427 (PMC3058098; doi:10.1371/journal.pmed.1000427)
Supplement: Table S2 — Mortality by HIV status and potential explanatory factors. (0.09 MB PDF) [file pmed.1000427.s002.pdf]

Table S2: Mortality by HIV status and potential explanatory factors.

Factor	Level	HIV negative			HIV positive		
		n	Deaths	Mortality rate per 100 pyo (95% CI)	n	Deaths	Mortality rate per 100 pyo (95% CI)
Overall		148	16	5.9 (3.6 ; 9.6)	140	51	21.4 (16.2 ; 28.1)
Age (years)	< 30	84	9	5.6 (2.9 ; 10.7)	43	14	19.7 (11.6 ; 33.2)
	30-40	37	3	4.4 (1.4 ; 13.6)	67	21	17.1 (11.2 ; 26.3)
	> 40	27	4	9.5 (3.6 ; 25.4)	30	16	35.5 (21.7 ; 57.9)
Sex	Female	29	5	10.9 (4.5 ; 26.1)	64	24	22.1 (14.8 ; 33.0)
	Male	119	11	4.9 (2.7 ; 8.8)	76	27	20.8 (14.2 ; 30.3)
Body mass index (kg/m^2)	< 15	21	4	10.3 (3.9 ; 27.5)	14	5	26.3 (10.9 ; 63.2)
	15-18.5	66	4	3.0 (1.1 ; 8.1)	80	32	24.2 (17.1 ; 34.2)
	> 18.5	59	7	7.1 (3.4 ; 14.9)	45	13	15.0 (8.7 ; 25.9)
Reason for TB retreatment	Relapse	67	7	6.0 (2.8 ; 12.5)	83	33	23.6 (16.8 ; 33.2)
	Failure	6	3	40.4 (13.0 ; 125.2)	3	3	186 (60 ; 576)
	Defaulter	75	6	4.1 (1.8 ; 9.1)	54	15	15.4 (9.3 ; 25.6)
Duration of TB symptoms (months)	< 3	93	5	2.8 (1.2 ; 6.8)	102	36	19.8 (14.3 ; 27.5)
	3–6	35	7	10.7 (5.1 ; 22.5)	26	10	22.2 (11.9 ; 41.2)
	> 6	20	4	12.7 (4.8 ; 33.8)	12	5	40.8 (17.0 ; 98.0)
Number of previous TB episodes	1	128	10	4.2 (2.3 ; 7.8)	114	45	23.4 (17.4 ; 31.3)
	2	15	4	14.6 (5.5 ; 38.8)	19	5	14.7 (6.1 ; 35.2)
	≥3	5	2	28.1 (7.0 ; 112.5)	7	1	8.3 (1.2 ; 58.7)
Time since most recent TB episode (months)	< 9	27	3	5.6 (1.8 ; 17.3)	30	9	19.3 (10.0 ; 37.0)
	9 – 15	48	6	6.9 (3.1 ; 15.4)	34	12	19.3 (11.0 ; 34.1)
	15 – 30	35	2	3.2 (0.8 ; 12.6)	33	13	23.4 (13.6 ; 40.3)
	>30	38	5	7.3 (3.1 ; 17.6)	43	17	22.8 (14.2 ; 36.7)

Resistance status	Sensitive H and R	118	8	3.5 (1.7 ; 7.0)	116	38	17.9 (13.0 ; 24.6)
	Resistant H or R	19	2	6.4 (1.6 ; 25.7)	17	7	32.4 (15.4 ; 68.0)
	MDR	11	6	50.4 (22.6 ; 112.2)	7	6	125 (56 ; 278)
Extent of disease on chest X-ray	Normal/Minimal	7	1	10.7 (1.5 ; 76.0)	25	9	23.0 (12.0 ; 44.3)
	Moderate	29	2	3.9 (1.0 ; 15.5)	48	17	20.8 (12.9 ; 33.5)
	Severe	111	13	6.3 (3.6 ; 10.8)	63	22	19.4 (12.8 ; 29.5)
Cavitation	No	26	2	5.5 (1.4 ; 21.8)	66	27	25.5 (17.5 ; 37.2)
	Yes	107	14	6.0 (3.6 ; 10.2)	70	21	16.4 (10.7 ; 25.1)
Sputum AFB smear grade	1+ / 2+	10	4	38.9 (14.6 ; 103.6)	20	8	26.1 (13.0 ; 52.2)
	3+	19	0	0	21	6	14.9 (6.7 ; 33.1)
	4*	119	12	5.4 (3.1 ; 9.5)	99	37	22.1 (16.0 ; 30.4)
DTP	≥ 7 days	40	4	6.0 (2.3 ; 16.0)	40	13	18.3 (10.6 ; 31.5)
	< 7 days	108	12	5.8 (3.3 ; 10.3)	100	38	22.7 (16.5 ; 31.1)
TB treatment adherence	Mostly adherent[1]	114	7	3.2 (1.5 ; 6.7)	103	32	16.8 (11.9 ; 23.8)
	Missed half or more	30	6	11.4 (5.1 ; 25.3)	29	11	23.3 (12.9 ; 42.1)
	No Info	4	3	891 (287 ; 2800)	8	8	605 (303 ; 1210)
CD4[2] (cells/ microL) and ART group[3]	CD4 ≥ 200	–	–	–	49	12	11.8 (6.7 ; 20.9)
	CD4 50-199, no ART				18	9	20.9 (10.9 ; 40.2)
	CD4 50-199, ART				26	3	7.3 (2.3 ; 22.5)
	CD4< 50, no ART				27	21	84.3 (55.0 ; 129.3)
	CD4< 50, ART				19	5	17.8 (7.4 ; 42.8)

BMI=Body mass index H=Isoniazid R=Rifampicin MDR= Multi-drug resistant

AFB=Acid fast bacilli DTP=Days to positive culture (BACTEC) ART=Antiretroviral therapy

[1] Category includes subjects that were fully adherent with treatment and those that missed a few doses.

[2] One HIV-infected subject did not have CD4 result.

[3] This is a time varying covariate; numbers are those for whom the category was their final category.
